# Safety and Efficacy of Bivalirudin versus Unfractionated Heparin Monotherapy in Patients with CAD and DM Undergoing PCI: A Retrospective Observational Study

**DOI:** 10.1155/2022/5352087

**Published:** 2022-11-30

**Authors:** Jing Li, Sanbao Chen, Sicong Ma, Mingque Yang, Zizhao Qi, Kun Na, Miaohan Qiu, Yi Li, Yaling Han

**Affiliations:** ^1^The Graduate School of Harbin Medical University, Harbin, China; ^2^Department of Cardiology, General Hospital of Northern Theater Command, Shenyang, Liaoning Province, China

## Abstract

**Introduction:**

Optimal anticoagulants for patients with diabetes mellitus (DM) undergoing percutaneous coronary intervention (PCI) are unclear. This retrospective observational study is aimed at evaluating efficacy and safety of bivalirudin versus unfractionated heparin (UFH) monotherapy in patients with DM undergoing PCI.

**Methods:**

A total of 3890 diabetic patients receiving PCI in the General Hospital of Northern Theater Command were divided into the bivalirudin group (*n* = 869) and the UFH group (*n* = 3021) according to different anticoagulant therapy regimens. Indication for PCI was in accordance with current guidelines including national cardiovascular data registry. The primary endpoint was 30-day net adverse clinical events (NACEs). The secondary endpoints included 30-day major adverse cardiac and cerebral events (MACCEs), bleeding events defined according to the Bleeding Academic Research Consortium (BARC) definition, and stent thrombosis (ST). Patients were matched by propensity score at a ratio of 1 : 1.

**Results:**

After propensity score matching, the bivalirudin group was associated with a lower incidence of NACEs (3.0% vs. 6.0%, *P* = 0.003) than the UFH group. The incidence of MACCE (1.7% vs. 3.3%, *P* = 0.033) was significantly lower in the bivalirudin group, mainly due to a lower mortality rate (0.6% vs. 2.0%, *P* = 0.010). In addition, patients in the bivalirudin group had less bleeding (1.4% vs. 3.0%, *P* = 0.022) than those in the UFH group, although BARC 2, 3, and 5 bleeding (0.1% vs. 0.6%, *P* = 0.218) was numerically lower.

**Conclusion:**

In diabetic patients undergoing PCI, bivalirudin was significantly associated with reduced risks of 30-day NACE and MACCE, mainly driven by the lower rates of bleeding and mortality, compared with heparin monotherapy.

## 1. Introduction

Although patients with diabetes mellitus (DM) and coronary artery disease (CAD) have an increased risk for ischemic events such as myocardial infarction (MI) and stent thrombosis (ST) compared to patients without DM [1, 2], previous studies have also demonstrated a higher rate of bleeding in this population, which is known to be significantly associated with mortality [3–5]. Due to the dilemma of balancing ischemic and bleeding risks, it is a challenge to determine antithrombotic strategies during PCI procedure in patients with DM.

The optimal anticoagulation strategy with favorable efficacy and tolerable bleeding risk has not been established for patients with DM undergoing PCI. Bivalirudin is associated with a lower risk of bleeding than unfractionated heparin (UFH) therapy in several trials [6–9]. However, several randomized controlled trials and meta-analyses showed that bivalirudin increases the risk of stent thrombosis (ST) in comparison with heparin in patients undergoing PCI, which arises the controversy in periprocedural anticoagulation [10–12]. Moreover, these trials mainly compared bivalirudin with UFH plus glycoprotein IIb/IIIa inhibitors (GPIs), while concomitant use of GPIs is considered only for bail-out in current guidelines because of the high risk of bleeding [13, 14]. Currently, evidence comparing bivalirudin with UFH monotherapy is limited, especially among diabetic patients undergoing PCI. Therefore, we performed an observational study evaluating the efficacy and safety of bivalirudin monotherapy versus heparin monotherapy in patients with DM undergoing PCI in a real-world cohort.

## 2. Materials and Methods

### 2.1. Study Population

This single-center, observational study recruited the consecutive patients with DM and CAD who underwent PCI from January 2016 to November 2018 in the Department of Cardiology, General Hospital of Northern Theater Command, Shenyang, China. Indication for PCI was in accordance with national cardiovascular data registry, which includes (1) elective; (2) urgent (required during same hospitalization to minimize further clinical deterioration, worsening or sudden chest pain, congestive heart failure, acute MI, anatomy, intra-aortic balloon pump, unstable angina with intravenous nitroglycerin, or angina at rest); (3) emergency (to procedure or in transit to the catheterization laboratory, ongoing ischemia despite maximal medical therapy, acute MI ≤24 hours before procedure, pulmonary edema requiring intubation, or shock with or without circulatory support); or (4) salvage (undergoing CPR enroute to PCI). The inclusion criteria were as follows: (1) age ≥ 18; (2) DM treated with insulin and/or oral agents; (3) at least one stent implemented; and (4) usage of bivalirudin or UFH during the procedure. The exclusion criteria were as follows: (1) administration of both bivalirudin and UFH during PCI; (2) the use of low-molecular-weight heparin; (3) cardiogenic shock; (4) aortic dissection; or (5) planned secondary PCI within 30 days. This study was approved by the Ethics Committee of General Hospital of Northern Theater Command, and an exemption for informed consent was approved simultaneously.

### 2.2. Medications

Bivalirudin was administered as a bolus of 0.75 mg/kg before the procedure, followed by infusion of 1.75 mg/kg/hour during the procedure and prolonged infusion at the PCI dose for at least 30 minutes after the procedure. For patients who used UFH, a bolus of 80~100 U/kg was administered. The activated clotting time (ACT) was measured using a Haemotec system 5 minutes after the bolus. An additional bivalirudin (0.3 mg/kg) or UFH (20 U/kg) bolus was given if the ACT was less than 225 seconds in the bivalirudin group or 200 seconds in the UFH group, respectively. GPIs were used only for bail-out only if there was evidence for no-reflow or a thrombotic complication. Antiplatelet therapy with aspirin and a P2Y12 inhibitor loading was regularly prescribed according to concurrent guidelines. After the procedure, all patients received clopidogrel (75 mg/day) or ticagrelor (180 mg/day) in combination with aspirin (100 mg/day) for at least 12 months. Other medications were given in the physician's direction according to standard local practice.

### 2.3. Data Collection and Follow-Up

Baseline clinical characteristics, laboratory tests, medications, and procedural characteristics were obtained from the hospital information system. Data were screened upon entry, and inaccurate data were checked, corrected, or cleared up. All participants were regularly followed up via centralized telephone or e-mail by trained personnel at 1, 6, and 12 months after the procedure and patients who could not be contacted over three times were considered lost to follow-up. Thirty-day clinical outcomes were extracted from the follow-up database. All clinical events were adjudicated by three experienced cardiologists who were blinded to the treatment allocations.

### 2.4. Study Endpoints

The primary endpoint was net adverse clinical events (NACEs) at 30 days after the procedure, defined as a composite of death, MI, stroke, urgent target lesion revascularization (uTLR), or any bleeding. The secondary endpoints were major adverse cardiovascular and cerebral events (MACCEs) at 30 days, defined as a composite of death, MI, stroke, and uTLR. MI was defined based on the Fourth Universal Definition of Myocardial Infarction Guidelines [15]. Stroke was defined as local or systemic loss of neurologic function attributable to a central nervous system vascular cause lasting for at least 24 h, documented by a CT scan or magnetic resonance imaging (MRI) or autoptic evidence. Other secondary endpoints included ST and any bleeding. Bleeding was defined according to Bleeding Academic Research Consortium (BARC) criteria [16], and ST was defined in accordance with the Academic Research Consortium definitions [17].

### 2.5. Statistical Analysis

Continual variables are presented as the mean ± standard deviation (SD) and were compared using the Student *t* test. Categorical data are presented as counts (%) and were compared using the *χ*2 test or Fisher's exact test, as appropriate. To minimize the selection bias, propensity score matching (PSM) was conducted. Patients were matched at a ratio of 1 : 1 between the bivalirudin and UFH groups using nearest-neighbor matching. Variables matched by propensity score included age, sex, smoking status, drinking status, hypertension, history of stroke, MI, PCI and coronary artery bypass graft (CABG), peripheral arterial disease (PAD), clinical presentation, estimated glomerular filtration rate (eGFR), left ventricular ejection fraction (LVEF), level of hemoglobin (Hb), platelet count, CRUSADE score, medications at discharge, radial or femoral access, target vessels, chronic total occlusion, length, diameter and number of stents, and SYNTAX score. The incidences of NACE, MACCE, bleeding, and death were compared between the two groups using Kaplan-Meier curves and tested by log-rank tests both before and after PSM. A two-tailed *p* value less than 0.05 was considered to be statistically significant. Statistical analyses were performed using IBM SPSS (version 22).

## 3. Results and Discussion

### 3.1. Baseline Characteristics

**Figure 1:**
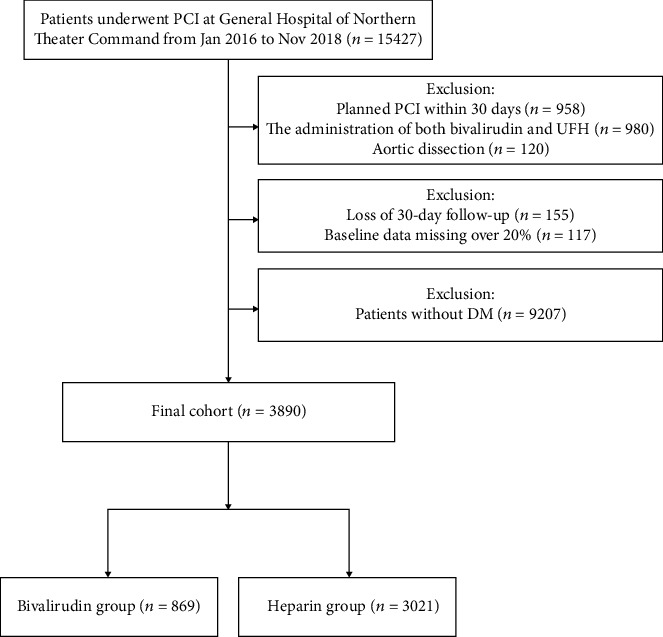
Patient flow chart for the study cohort.

Among 15,427 patients undergoing PCI from January 2016 to November 2018 in the Department of Cardiology, General Hospital of Northern Theater Command, 3890 patients with DM were enrolled in our study (Figure 1). A total of 869 (22.3%) patients received bivalirudin and 3021 (77.7%) patients received UFH during the perioperative period. Most patients (94.0%) received PCI via a transradial approach, and 1406 (36.1%) patients presented with acute MI (Table 1). Compared with the UFH group, patients in the bivalirudin group were older, more likely to be female, and have a history of prior stroke. The proportion of ST-segment elevation myocardial infarction was higher in the bivalirudin group than in the UFH group. In addition, eGFR, level of Hb, platelet count, and the ratio of radial access were lower and CRUSADE scores were higher in patients with bivalirudin than in those with UFH. The proportion of use of GPIs in the two groups was 15.1% and 13.3%, respectively (*P* = 0.192). After PSM, baseline characteristics, including the proportion of GPI administration (13.6% vs. 13.3%, *P* = 0.888), were well balanced between the two groups.

**Figure 2:**
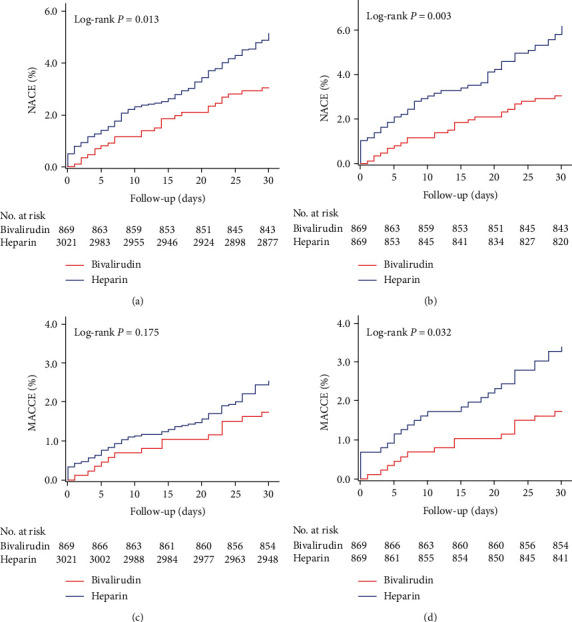
Kaplan-Meier curves of NACE and MACCE.  Figure 2 shows the Kaplan-Meier curves for NACE (a, b) and MACCE (c, d). Before propensity score matching (a, c); after propensity score matching (b, d). NACE: net adverse clinical event; MACCE: major adverse cardiovascular and cerebral event.

**Figure 3:**
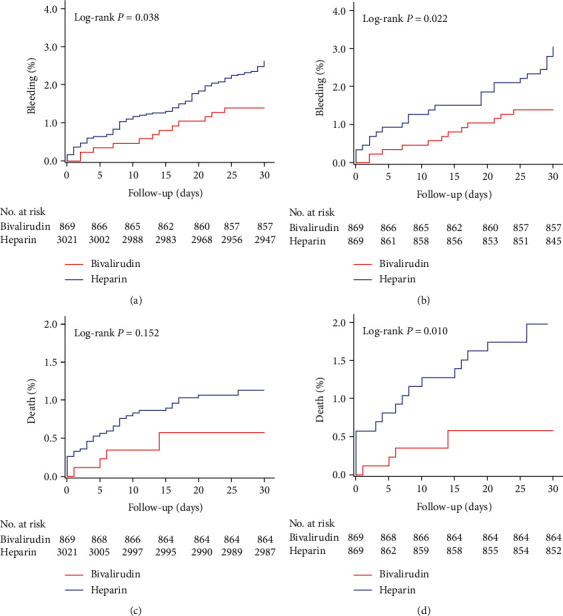
Kaplan-Meier curves of BARC-defined any bleeding and death.  Figure 3 shows the Kaplan-Meier curves for BARC-defined any bleeding (a, b) and death (c, d). Before propensity score matching (a, c); after propensity score matching (b, d). BARC: bleeding academic research consortium.

At 30 days, the primary endpoint, NACE, occurred in 26 (3.0%) patients in the bivalirudin group and 151 (5.0%) patients in the UFH group (Table 2). The risk of NACE was significantly lower in the bivalirudin group than in the UFH group irrespective of whether PSM was performed (before PSM: 3.0% vs. 5.0%, *P* = 0.012; after PSM: 3.0% vs. 6.0%, *P* = 0.003, Figures 2(a) and 2(b)). The significant difference was driven by both the lower rates of BARC-defined any bleeding (before PSM: 1.4% vs. 2.6%, *P* = 0.038; after PSM: 1.4% vs. 3.0%, *P* = 0.022, Figures 3(a) and 3(b)) and the reduction of MACCE (before PSM: 1.7% vs. 2.5%, *P* = 0.175; after PSM: 1.7% vs. 3.3%, *P* = 0.033, Figures 2(c) and 2(d)).

The lower incidence of MACCEs was mainly ascribed to a significantly lower rate of death (before PSM: 0.6% vs. 1.1%, *P* = 0.151; after PSM: 0.6% vs. 2.0%, *P* = 0.033, Figures 3(c) and 3(d)) in the bivalirudin group than in the UFH group. The rates of MI, stroke, and uTLR were similar between the two groups. In terms of the safety endpoint, although without significant difference, the rate of BARC type 2, 3, and 5 bleeding in the bivalirudin group was quantitatively lower than that in the heparin group (before PSM: 0.1% vs. 0.3%, *P* = 0.577; after PSM: 0.1% vs. 0.6%, *P* = 0.218). The 30-day ST occurred in only 2 patients in each group, including 2 definite ST (0.1%) in heparin group and 2 probable ST (0.2%) in bivalirudin group. No statistically significant difference in the rate of ST was found between the two groups (before PSM: 0.2% vs. 0.1%, *P* = 0.218; after PSM: 0.2% vs. 0.1%, *P* = 1.000) (Table 2). No interation in the rate of NACE, MACCE, or all bleeding was found between CKD or no-CKD (defined as eGFR < 60 mL/min/1.73 m^2^), or between transradial or transfemoral approach (*P* for interation > 0.05 for both, Supplemental Materials Table S1).

## 4. Discussion

The main finding of this real-world observational study was that in patients with DM undergoing PCI, an antithrombotic regimen of bivalirudin monotherapy, compared with UFH monotherapy (without planned use of a GPI), was associated with a lower incidence of NACE. This finding was mainly ascribed to a significant reduction in bleeding and MACCE in the bivalirudin group. Regarding MACCE, patients in the bivalirudin group had a significantly lower mortality rate than those in the UFH group. Although there was a lower bleeding rate in the bivalirudin group, the incidence of BARC 2, 3, and 5 bleeding was just numerically lower than that in the UFH group.

The present study demonstrated that the benefit of MACCE in the bivalirudin group was mainly due to a significantly lower mortality at 30 days. In a post hoc analysis of HORIZONS-AMI, the rate of cardiac death was significantly lower in diabetic patients treated with bivalirudin than in those treated with UFH plus a GPI [18]. Similarly, in a pooled analysis of the REPLACE-2, ACUITY, and HORIZONS-AMI trials, bivalirudin was associated with reduced 1-year mortality in patients with DM [19]. Moreover, the most recently published randomized trial, the BRIGHT-4 study, found that bivalirudin with a prolonged infusion significantly reduced the 30-day mortality of patients with ST-segment elevation myocardial infarction undergoing PCI in comparison with heparin monotherapy [20]. The lower rate of death associated with bivalirudin use in patients with DM may have several explanations. First, diabetic patients with CAD tend to have fibrin-rich thrombi and platelet dysfunction [21]. Moreover, hyperglycaemia-induced upregulation of glycoproteins and increased expression of platelet activation markers boost the progression of atherosclerosis, leading to higher mortality and ischemic events in patients with DM than in those without DM [22, 23], which might also influence the pharmacodynamics of antiplatelet agents. Second, heparin can directly bind to the platelet glycoprotein IIb/IIIa receptor and increase platelet reactivity, thereby multiplying the risk of ischemic events in patients with DM; conversely, bivalirudin can not only inhibit circulating and clot-bound thrombin but also thrombin-induced platelet activation, with antiplatelet and anti-inflammatory effects similar to those of UFH plus a GPI [24, 25].

Regarding bleeding events, previous studies comparing bivalirudin with UFH in combination with a GPI in patients with DM have generated conflicting results [18, 26, 27]. The post hoc analysis of the ACUITY trial demonstrated a decrease in major bleeding with bivalirudin use in diabetic patients undergoing PCI [26, 28]; whereas, subgroup analyses from the HORIZONS-AMI and NAPLES studies showed no differences in major bleeding between bivalirudin and UFH [18, 27]. A meta-analysis found that the benefit of decreased major bleeding with bivalirudin was only seen when a GPI was mandated in the heparin group [29]. A recent randomized trial, VALIDATE-SWEDEHEART, which compared bivalirudin with heparin monotherapy in MI, also showed no significant difference with respect to major bleeding events [30]. In our study, bivalirudin monotherapy was associated with significantly lower bleeding than UFH monotherapy. With regard to BARC 2, 3, and 5 bleeding, although no difference was found between the two groups, just as in the above study, the rate of BARC 2, 3, and 5 bleeding in the UFH group was six times as high as that in the bivalirudin group. Moreover, for patients with chronic kidney disease, who are deemed as a special population with a high bleeding risk [31], our subgroup analysis showed that bivalirudin did not significantly increase the rate of bleeding. Given the 30-day mortality and bleeding benefits, the results of our study suggest that bivalirudin might be a better anticoagulant in patients with DM undergoing PCI than UFH.

Clinical trials evaluating the efficacy of bivalirudin in patients undergoing primary PCI obtained inconsistent results regarding ST at 30 days [6, 11, 30, 32]. In the HEAT-PPCI trial, the rate of 28-day ST in the bivalirudin group was significantly higher than that in the heparin group, while in the BRIGHT and VALIDATE-SWEDEHEART trials, there were no significant differences in the rate of 30-day ST between bivalirudin and heparin [11, 30, 32]. Bivalirudin in the HEAT-PPCI trial was used for a short duration, while in the present study, prolonged infusion of bivalirudin was given at the PCI dose during the PCI procedure and for at least 30 minutes after the procedure. Consistent with our study, all patients receiving bivalirudin in the BRIGHT trial and over 65% of patients receiving bivalirudin in the VALIDATE-SWEDEHEART trial received a prolonged infusion, and prolonged infusion appeared beneficial in terms of ST [30, 32]. A subgroup analysis of the MATRIX study also demonstrated that prolonged bivalirudin infusion at a full dose (1.75 mg/kg/h for ≤4 h) was associated with a significantly lower ST than no infusion, irrespective of the type of acute coronary syndrome (ACS) [33]. A meta-analysis including 13,505 patients from pivotal randomized trials demonstrated that bivalirudin with full dose post-PCI infusion is superior to heparin monotherapy in preventing early ST and major bleeding in patients who underwent primary PCI [34]. In terms of ST reported from our study, the rates of ST in both group were extremely low (0.2% in bivalirudin group and 0.1% in heparin group). Therefore, no significant difference in our study was found between bivalirudin and heparin due to the limited sample size, which led to a low event number of ST. Recently, the newest BRIGHT-4 study with a total sample size of more than 6000 patients with STEMI showed that bivalirudin with a prolonged infusion post-PCI significantly decreased the rate of ST compared with heparin, indicating that bivalirudin has a substantial effect on preventing ST, to some extent, settled down this dispute [20].

Several limitations should be acknowledged in the present study. First, as an observational study, patients were not randomized according to antithrombotic strategy, and patients in the bivalirudin group were at higher risk of bleeding. Although PSM was performed, there may exist potential confounders between groups. Second, we only investigated 30-day clinical outcomes, and long-term follow-up will add further perspective. Third, patients who received heparin before the use of bivalirudin, a common phenomenon with potential benefits [30, 35, 36], were excluded per study protocol, which might limit the generalizability of our findings.

## 5. Conclusion

In this large, real-world cohort of patients with DM undergoing PCI, bivalirudin, compared with UFH monotherapy, was significantly associated with a reduction in 30-day NACE and MACCE, mainly driven by the significantly lower incidences of bleeding and mortality.

## Figures and Tables

**Table 1:** Baseline characteristics.

Variable	Before PSM			After PSM		
	Bivalirudin (*n* = 869)	Heparin (*n* = 3021)	*P* value	Bivalirudin (*n* = 869)	Heparin (*n* = 869)	*P* value
Age (year)	63.80 ± 9.91	60.99 ± 9.69	<0.001	63.80 ± 9.91	63.51 ± 9.35	0.524
Female	309 (35.6)	899 (29.8)	0.001	309(35.6)	310 (35.7)	0.960
Smoker	279 (32.1)	1145 (37.9)	0.002	279 (32.1)	306 (35.2)	0.171
Drinker	130 (15.0)	505 (16.7)	0.217	130 (15.0)	137 (15.8)	0.641
Hypertension	620 (71.3)	2124 (70.3)	0.554	620 (71.3)	651 (74.9)	0.093
Previous MI	180 (20.7)	656 (21.7)	0.527	180 (20.7)	175 (20.1)	0.766
Previous PCI	255 (29.3)	979 (32.4)	0.087	255 (29.3)	259 (29.8)	0.833
Previous CABG	14 (1.6)	51 (1.7)	0.876	14 (1.6)	18 (2.1)	0.475
Peripheral arterial disease	19 (2.2)	42 (1.4)	0.096	19 (2.2)	15 (1.7)	0.488
Clinical presentation						
SCAD	1 (0.1)	2 (0.1)	0.532	1 (0.1)	2 (0.2)	0.560
UA	511 (58.8)	1947 (64.4)	0.002	511 (58.8)	500 (57.5)	0.593
AMI	355(40.9)	1051(34.8)	0.001	355(40.9)	361(41.5)	0.770
NSTEMI	157 (18.1)	476 (15.8)	0.104	157 (18.1)	150 (17.3)	0.660
STEMI	198 (22.8)	575 (19.0)	0.015	198 (22.8)	211 (24.3)	0.462
eGFR (L·min^−1^·1.73 m^−1^)	100.14 ± 36.78	110.20 ± 39.87	<0.001	100.14 ± 36.78	103.22 ± 55.78	0.186
Hb (g/L,$\;\bar{x\pm \text{s}}$)	132.42 ± 17.44	137.77 ± 15.27	<0.001	132.42 ± 17.44	132.67 ± 16.65	0.764
Platelet count (10^9^/L)	218.03 ± 61.24	223.14 ± 58.32	0.028	218.0 ± 61.24	219.38 ± 55.06	0.637
LVEF (%)	58.75 ± 9.10	58.46 ± 9.16	0.435	58.75 ± 9.10	58.67 ± 8.95	0.851
CRUSADE score	34.00 ± 13.53	28.91 ± 10.12	<0.001	34.00 ± 13.53	33.34 ± 11.74	0.286
Antiplatelet therapy before PCI						
Aspirin	858 (98.7)	2992 (99.0)	0.431	858 (98.7)	859 (98.8)	0.826
Clopidogrel	789 (90.8)	2631 (87.1)	0.003	789 (90.8)	782 (90.0)	0.569
Ticagrelor	226 (26.0)	839 (27.8)	0.304	226 (26.0)	200 (23.0)	0.147
Medications at discharge						
Aspirin	842 (96.9)	2951 (97.7)	0.188	842 (96.9)	850 (97.8)	0.232
Clopidogrel	622 (71.6)	2084 (69.0)	0.143	622 (71.6)	635 (73.1)	0.486
Ticagrelor	237 (27.3)	887 (29.4)	0.231	237 (27.3)	220 (25.3)	0.354
Statin	812 (93.4)	2788 (92.3)	0.254	812 (93.4)	813 (93.6)	0.922
ACEI	435 (50.1)	1533 (50.7)	0.721	435 (50.1)	436 (50.2)	0.962
ARB	182 (20.9)	569 (18.8)	0.165	182 (20.9)	172 (19.8)	0.551
*β*-Blocker	657 (75.6)	2242 (74.2)	0.407	657 (75.6)	646 (74.3)	0.542
CCB	235 (27.0)	695 (23.0)	0.014	235 (27.0)	227 (26.1)	0.664
Radial access	799 (91.9)	2859 (94.6)	0.003	799 (91.9)	818 (94.1)	0.073
Target vessel						
LM	42 (4.8)	157 (5.2)	0.668	42 (4.8)	38 (4.4)	0.647
LAD	341 (39.2)	1205 (39.9)	0.731	341 (39.2)	341 (50.0)	1.000
LCX	188 (21.6)	652 (21.6)	0.974	188 (21.6)	166 (19.1)	0.190
RCA	270 (31.1)	944 (31.2)	0.921	270 (31.1)	280 (32.2)	0.606
CTO (%)	22 (2.5)	78 (2.6)	0.934	22 (2.5)	24 (2.8)	0.765
Syntax score	11.9 ± 8.4	12.0 ± 8.3	0.826	11.9 ± 8.4	11.8 ± 8.3	0.716
Length of stents	43.07 ± 28.16	42.46 ± 28.23	0.573	43.07 ± 28.16	42.83 ± 27.51	0.856
Diameter of stents	2.99 ± 0.66	3.01 ± 1.24	0.574	2.97 ± 0.37	3.01 ± 1.24	0.302
Number of stents	1.58 ± 0.93	1.60 ± 0.94	0.476	1.59 ± 0.91	1.60 ± 0.94	0.717
Use of GPIs	457 (15.1)	116 (13.3)	0.192	118 (13.6)	116 (13.3)	0.888

PSM: propensity score matching; MI: myocardial infarction; PCI: percutaneous coronary intervention; CABG: coronary artery bypass graft; SCAD: stable coronary artery disease; UA: unstable angina; NSTEMI: non-ST-segment elevation myocardial infarction; STEMI: ST-segment elevation myocardial infarction; eGFR: estimated glomerular filtration rate; Hb: hemoglobin; LVEF: left ventricular ejection fraction; ACEI: angiotensin converting enzyme inhibitor; ARB: angiotensin receptor antagonist. CCB: calcium channel blocker. LM: left main; LAD: left anterior descending; LCX: left circumflex; RCA: right coronary artery; CTO: chronic total occlusion; GPIs: glycoprotein IIb/IIIa inhibitors. Data are presented as count (%) or mean ± standard deviation.

**Table 2:** 30-day clinical outcomes.

Variable	Before PSM			After PSM		
	Bivalirudin (*n* = 869)	Heparin (*n* = 3021)	*P* value	Bivalirudin (*n* = 869)	Heparin (*n* = 869)	*P* value
NACE	26 (3.0)	151 (5.0)	0.012	26 (3.0)	52 (6.0)	0.003
MACCE	15 (1.7)	76 (2.5)	0.175	15 (1.7)	29 (3.3)	0.033
Death	5 (0.6)	34 (1.1)	0.151	5 (0.6)	17 (2.0)	0.010
Nonfatal MI	0	1 (0)	1.000	0	1 (0)	1.000
Nonfatal stroke	3 (0.3)	4 (0.1)	0.395	3 (0.3)	2 (0.2)	1.000
uTLR	7 (0.8)	38 (1.3)	0.272	7 (0.8)	10 (1.2)	0.465
Bleeding						
All bleedings	12 (1.4)	78 (2.6)	0.038	12 (1.4)	26 (3.0)	0.022
BARC 2, 3, and 5 bleedings	1 (0.1)	9 (0.3)	0.577	1 (0.1)	5 (0.6)	0.218
BARC 2 bleedings	1 (0.1)	8 (0.3)	0.694	1 (0.1)	4 (0.5)	0.374
BARC 3 and 5 bleeding	0	1 (0)	1.000	0	1 (0.1)	1.000
Stent thrombosis	2 (0.2)	2 (0.1)	0.218	2 (0.2)	1 (0.1)	1.000
Definite	0	2 (0.1)	1.000	0	1 (0.1)	1.000
Probable	2 (0.2)	0	0.050	2 (0.2)	0	0.500

NACE: net adverse clinical event; MACCE: major adverse cardiovascular and cerebral event; MI: myocardial infarction; uTLR: urgent target lesion revascularization; BARC: bleeding academic research consortium. Data are presented as count (%).

## Data Availability

The data in this study is available with reasonable requests by contacting the corresponding authors.

## Additional Points

*Key Summary Points*. Why carry out this study? Previous trials mostly compared bivalirudin with UFH plus GPI, while concomitant use of GPI is considered only for bail-out in current guidelines account of high risk of bleeding. Currently, evidences comparing bivalirudin with UFH monotherapy are limited, especially among diabetic patients undergoing PCI. What was learned from the study? This large-sample, observational study found that in diabetic patients undergoing PCI, bivalirudin was significantly associated with a significantly lower risk of 30-day NACE, driven by a lower bleeding and MACCE, and a significantly lower mortality, compared with heparin monotherapy. What were the study conclusions? In addition to improving our understanding of the precise effects of bivalirudin and heparin in contemporary clinical practice, these findings emphasize the benefit of bivalirudin versus heparin monotherapy in reducing 30-day mortality in diabetic patients.

## Supplementary Materials

Supplementary Materials Table S1 was shown in the supplemental materials.
