# Supplementary material for: Safety and Efficacy of Bivalirudin versus Unfractionated Heparin Monotherapy in Patients with CAD and DM Undergoing PCI: A Retrospective Observational Study
Source: Cardiovasc Ther. 2022 Nov 30;2022:5352087. doi: 10.1155/2022/5352087 (PMC9729030; doi:10.1155/2022/5352087)
Supplement: Supplementary Materials — Table S1 was shown in the supplemental materials. [file 5352087.f1.docx]

Supplemental Materials

Table S1. Subgroup analysis of bivalirudin versus heparin via Cox risk regression

|  | NACE | P for interaction | MACCE | P for interaction | All bleeding | P for interaction |
| --- | --- | --- | --- | --- | --- | --- |
| Trans-radial | 0.52 (0.30-0.90) | 0.34 | 0.95 (0.70-1.28) | 0.87 | 0.61 (0.39-0.96) | 0.60 |
| Trans-femoral | 0.77 (0.26-2.26) |  | 0.82 (0.45-1.45) |  | 0.56 (0.33-0.93) |  |
| eGFR < 60ml/min/1.73m^2^ | 0.58 (0.34-1.00) | 0.57 | 0.96 (0.89-1.07) | 0.67 | 0.51 (0.27-0.94) | 0.09 |
| eGFR ≥ 60ml/min/1.73m^2^ | 0.93 (0.64-1.37) |  | 0.81 (0.41-1.60) |  | 0.82 (0.45-1.90) |  |

NACE: net adverse clinical event; MACCE: major adverse cardiovascular and cerebral event.
